# Theory of Choice in Bandit, Information Sampling and Foraging Tasks

**DOI:** 10.1371/journal.pcbi.1004164

**Published:** 2015-03-27

**Authors:** Bruno B. Averbeck

**Affiliations:** Laboratory of Neuropsychology, National Institute of Mental Health, National Institutes of Health, Bethesda, Maryland, United States of America; University of Minnesota, United States of America

## Abstract

Decision making has been studied with a wide array of tasks. Here we examine the theoretical structure of bandit, information sampling and foraging tasks. These tasks move beyond tasks where the choice in the current trial does not affect future expected rewards. We have modeled these tasks using Markov decision processes (MDPs). MDPs provide a general framework for modeling tasks in which decisions affect the information on which future choices will be made. Under the assumption that agents are maximizing expected rewards, MDPs provide normative solutions. We find that all three classes of tasks pose choices among actions which trade-off immediate and future expected rewards. The tasks drive these trade-offs in unique ways, however. For bandit and information sampling tasks, increasing uncertainty or the time horizon shifts value to actions that pay-off in the future. Correspondingly, decreasing uncertainty increases the relative value of actions that pay-off immediately. For foraging tasks the time-horizon plays the dominant role, as choices do not affect future uncertainty in these tasks.

## Author Summary

Numerous choice tasks have been used to study decision processes. Some of these choice tasks, specifically n-armed bandit, information sampling and foraging tasks, pose choices that trade-off immediate and future reward. Specifically, the best choice may not be the choice that pays off the highest reward immediately, and exploration of unknown options vs. exploiting known options can be a normatively useful strategy. We characterized the optimal choice strategies across these tasks using Markov Decision Processes (MDPs). The MDP framework can characterize optimal choice strategies when choices are affected by the value of future rewards. We found that uncertainty and time horizon have important effects on the choice strategies in these tasks. Specifically, in bandit and information sampling tasks, increasing uncertainty increases the value of exploring choice options that tend to pay off in the future, while decreasing uncertainty increases the value of choice options that pay off immediately. These effects are increased when time horizons are longer. Foraging tasks differ in that uncertainty plays a minimal role. However, time horizon is still important in foraging. Specifically, for long time horizons, travel delays to rewards become less relevant.

## Introduction

Decision making has been studied with a wide array of tasks. Choices in many of these tasks either do not affect future choices or are modeled as if they do not affect future choices. For example, when asked to choose between gambles (e.g. 50% chance of $20 or 100% chance of $11), the choice in the current trial does not affect the gambles presented in the next trial, or the information on which one decides in the next trial. Correspondingly, even reinforcement learning tasks, where choices do affect the information that will be available for future choices, are often modeled using delta rule reinforcement learning (DRRL) or logistic regression, neither of which provides a normative description of the task. These modeling approaches assume that current choices should be driven entirely by past outcomes without considering how they will affect the future.

Many interesting decision making problems, however, require consideration of how current choices will affect the future [1–7]. For example, there has been interest in the explore-exploit tradeoff [8–17], information sampling [4,6], and foraging [18,19]. Explore-exploit trade-offs exist in any real-world decision making context where one has to choose between continuing to exploit a known option, for example a familiar restaurant, vs. exploring an unknown or novel restaurant. Similarly, information sampling underlies many deliberative choice processes where one collects information before committing to a decision. For example, one might study product reviews or ratings before making a large purchase. These tasks require more sophisticated choice strategies because choices can be driven by future expected values. In other words, the best choice may not be the one that delivers the largest immediate reward. The best choice may lead to larger rewards in the future at the expense of smaller immediate rewards. Choices in these tasks can be modeled with markov decision processes (MDPs). MDPs provide a general modeling framework, useful in tasks where the future depends upon what one chooses in the present. If one assumes that an agent is maximizing the expected (discounted or undiscounted) total reward, MDPs can be used to provide normative, or at least approximately normative, solutions to most current decision problems.

While the choice behavior of subjects often deviates from normative behavior [4], particularly in patient groups [5,20,21], normative models are still important. Specifically, normative models identify the information on which decisions should be based, and the computations that must be carried out on that information. These two points can be conceptualized as the strategy optimal for the task. Further, normative models can be parameterized to fit the behavior of individual subjects [4,5,22]. This approach can provide insight into how subjects are deviating from the normative model and therefore it can suggest specific deficits or biases, as opposed to an overall change in task performance.

Here, we used MDPs to model n-armed bandit, information sampling, and foraging tasks. Normative solutions to some of these tasks, to our knowledge, do not currently exist in the literature. There is, however, a long theoretical literature on binary bandit tasks [23,24], and some foraging tasks have been modeled using the marginal value theorem [25]. For MDPs, the development of approximation techniques using basis functions has opened up the solution of a much larger class of problems than was tractable previously [26]. The normative solutions provide insight into the optimal strategies. We also used the models to examine several specific questions. For example, when is it useful to explore in a bandit task, and which features of the task can increase the value of exploring? How can non-stationarity drive exploration? Furthermore, once the tasks were mapped into the MDP framework we could examine their similarities and differences. This showed that decisions in all of these tasks pose a trade-off between immediate and future expected rewards. Further, we identified two factors that are important to this trade-off in these tasks. The first is uncertainty and the second is the time horizon. In bandit and information sampling tasks future expected values are relatively higher for options about which there is more uncertainty. When there is less uncertainty, action values are driven more by immediate expected reward. Further, uncertainty, and the value of exploring uncertain options is more valuable when the time horizon is longer. We also show that reward rate maximization in foraging tasks with an undiscounted, infinite time horizon is insensitive to travel delays to patches. In general with MDPs, infinite horizon undiscounted models are insensitive to finite delays to rewards.

## Results

We used markov decision processes, either partially observed (POMDPs) or fully observed (MDPs) to model several choice tasks. For these models we are interested in the utility, u_t_. For MDPs the utility depends on the state, and for POMDPs it depends on the information state. We indicate both states and information states by s_t_. The utility is then given by the action, a, that maximizes action value, Q(s_t,_a):

$$u_{t}\left(s_{t}\right)\;=\;\underset{a\in A_{s_{t}}}{\max}\left\{Q(s_{t},a)\right\}$$

The action value:

$$Q\left(s_{t},a\right)\;=\;r\left(s_{t},a\right)+C(s_{t},a)\;+\gamma \sum_{j\in S}p(j|s_{t},a)u_{t+1}(j)$$

can be broken down into the immediate: r(s_t_,a)+C(s_t_,a) and future: γΣ_j∊s_p(j|s_t_,a)u_t+1_(j) expected values, which we will call IEV and FEV respectively. The IEV is the expected reward, r(s_t_,a), for taking action a in state s_t_ plus a possible cost to sample, C(s_t_,a). These occur immediately. The FEV is the expected value of the utility of the next state, where the state transition function or the probability of transitioning to state j when taking action a in state s_t_, is p(j|s_t_,a). The FEV is an estimate of the (possibly) discounted future rewards that will be obtained, given the current action. The state is the information on which decisions are based. For most of the tasks, except the foraging tasks, the state is a hidden variable, and this hidden variable gives rise to observations through an observation model. In this case, one is dealing with a partially observable Markov Decision Process (POMDP). The observations define the information state, and one can infer the value of the hidden state using the observations. We explicitly refer to the state as the information state for POMDPs.

### Exploration in a stationary two-armed bandit

When the environment is unknown, and model-free reinforcement learning (RL) is used to learn the environment [27], exploration can be used to drive the RL algorithm to sample from the complete space of possible options. Here we deal with tasks where the environment is specified and MDPs (or POMDPs) can be used to calculate expected values for each state. Therefore heuristic exploration does not have to be used to make choices. Exploration, if it is defined as selecting options which have a smaller IEV but a larger action value, however, can still be optimal [27]. If an agent is maximizing total expected reward, an option with a smaller IEV can be selected if its FEV is relatively larger. Thus, immediate rewards can be foregone to obtain more total rewards over the relevant time horizon. We began by examining the explore-exploit trade-off in a stationary 2-armed bandit task, in which both bandits paid-off with the same fixed reward. The bandits varied, however, in the fraction of times they delivered a reward if chosen. In this case the explore-exploit trade-off affects the first few choices, before both targets have been sampled a few times. We modeled this as a finite state, finite horizon, undiscounted POMDP, where the information states were the number of times each bandit was chosen, C_i_ and the number of times each bandit was rewarded, R_i_. This information state space is formed by the sufficient statistics for the two bandit processes. Transitions through the information state space occur after each choice and its associated outcome and they correspond to belief updates for the process.

**Fig 1:**
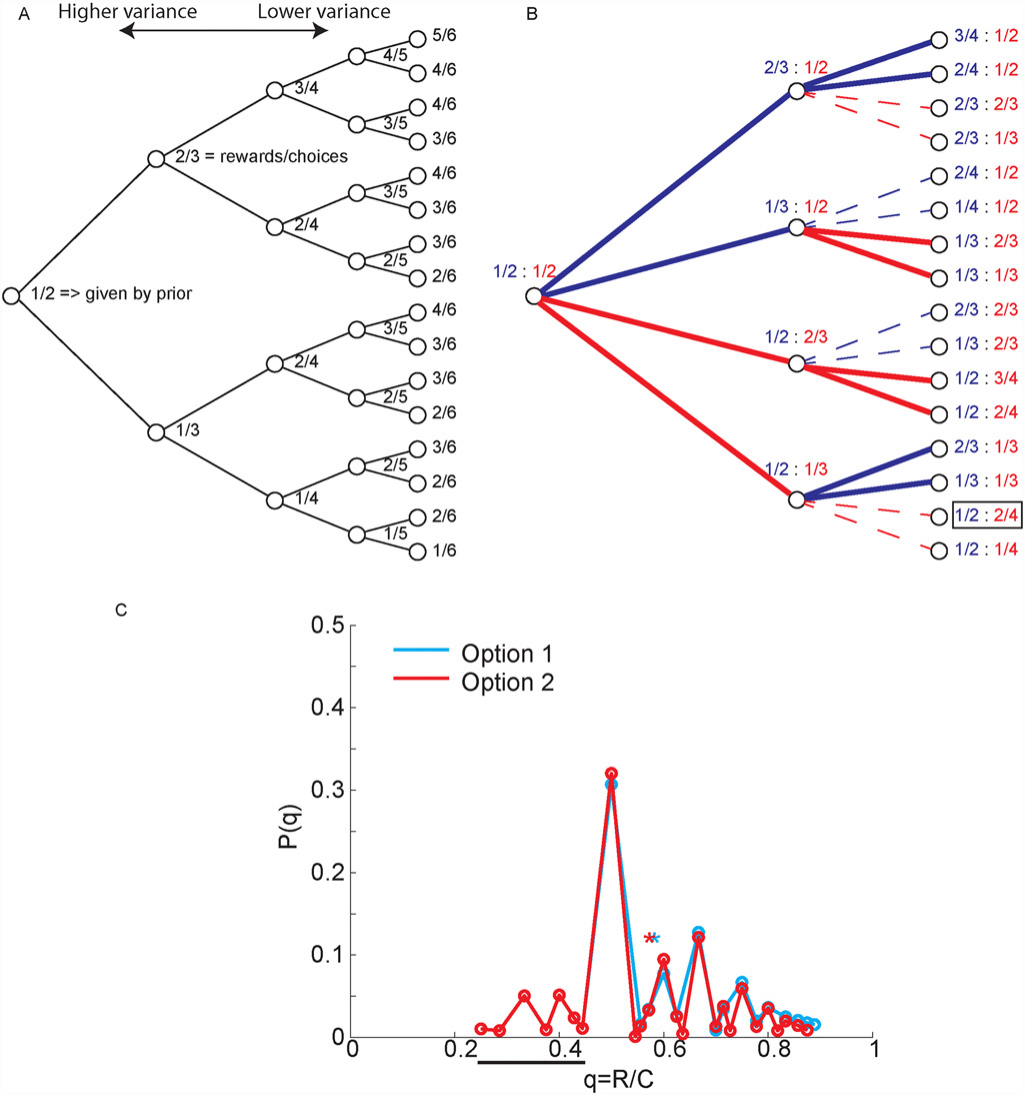
Bandit state space. A. A portion of the reward distribution tree, starting from a Beta(1,1) prior for one of the bandit options. As one of the options is chosen, the outcomes traverse this tree. The number at each node indicates the posterior over the number of rewards (numerator) and the number of times the option has been sampled (denominator). B. Product space across both bandit options. Blue lines (and fractions) indicate choice of option 1, red lines (and fractions) indicate choice of option 2. The numerator and denominator of the fractions are as in panel A and define the posterior probability of a reward. Thick lines show actions that would be taken from each node by an optimal policy, thin dashed lines show options that are not taken by an optimal policy. C. Distribution of reward probabilities (i.e. choices/rewards) over a finite horizon (N = 8 choices) starting from two different beta priors (Option 1: Beta(1,1) and Option 2: Beta(2,2)) which can be interpreted as different amounts of experience with the options. These priors correspond to being in the state 1/2:2/4 indicated in panel b with a box. The solid black bar under the x axis indicates q values for which p(q) is identical. Asterisks superimposed on the plots show the means of the two distributions (0.575 and 0.585 for option 2 and option 1 respectively).

To examine the information state space for the bandit task more quantitatively, we can examine the distributions over expected future reward values generated in the task. Each of the bandit options is represented by a tree of possible outcomes (Fig. 1A). Each node in the tree defines the information state (i.e. R_i_,C_i_) for that option. The information state can be used to estimate the underlying reward probability, q for each bandit option, where q is the hidden state of the system. As one of the options is sampled, the tree is traversed. With a binomial likelihood function and a beta(α,β) prior, the posterior over reward probability is given by

$$p\left(q|r_{i},c_{i}\right)\propto p\left(r_{i},c_{i}|q\right)p\left(q\right)$$

$$\text{p}(\text{q}|{\text{r}}_{\text{i}},{\text{c}}_{\text{i}})\propto {\text{q}}^{{\text{r}}_{\text{i}}}{\left(1-\text{q}\right)}^{{\text{c}}_{\text{i}}-{\text{r}}_{\text{i}}}{\text{q}}^{\alpha -1}{\left(1-\text{q}\right)}^{\beta -1}$$

$$p(q|r_{i},c_{i})\propto q^{r_{i}+\alpha -1}{\left(1-q\right)}^{c_{i}-r_{i}+\beta -1}$$

The beta prior is the natural conjugate prior for the binomial likelihood function. Therefore, the prior can be interpreted as data. The posterior expected value is $\langle q|R_{i},C_{i}\rangle \;=\;\frac{R_{i}}{C_{i}}\;=\;\frac{\alpha +r_{i}}{\alpha +\beta +c_{i}}$, where we have defined the actual choices and rewards as r_i_,c_i_, and the posterior choices and rewards as the data plus the prior R_i_ = r_i_ + α,C_i_ = c_i_ +α + β. If we start with a beta(α = 1,β = 1) prior we have posterior values of R_i_ = 1,C_i_ = 2 for each bandit arm before any options have been sampled (Fig. 1A). The possible posterior expected values are given by the nodes of the tree (Fig. 1A). These nodes are also the immediate expected value for a choice, i.e. <$r\left(s_{t},a\right)>\;=\;\frac{R_{i}}{C_{i}}$, and these values also define the transition probabilities. Thus, if one is in state, R_i_,C_i_ one transitions to R_i_+1,C_i_+1 with probability $p\left(j\;=\;R_{i}+1,C_{i}+1|s_{t}\;=\;R_{i},\;C_{i},a\right)\;=\;\frac{R_{i}}{C_{i}}$ and one transitions to R_i_,C_i_+1 with probability $p\left(j\;=\;R_{i},C_{i}+1|s_{t}\;=\;R_{i},\;C_{i},a\right)\;=\;1-\frac{R_{i}}{C_{i}}$. This defines two of the terms on the r.h.s. of equation 2 (ignoring the cost to sample). The other term on the r.h.s. of equation 2 is the utility of the next state, u_t+1_. These utilities are recursively related to future utilities, u_t+2_, etc. However, in the final trial, assuming a task where there are a finite number of trials and the number is known a-priori, there is no FEV because there will be no choices in the following trial. Therefore, utilities in the final trial, t = N, are given by the IEV, <r_t_(s_t_,a)>. The IEV for each state that can exist in the final trial can be directly calculated from these information states. Once these are calculated, one can calculate the utilities for t-1, and continue backwards until the utilities for the current trial can be calculated. This is the backwards induction algorithm ([28]; see methods).

When an option has not been sampled, any point in the tree can potentially be reached, although not under the optimal policy, and the distribution over reward probabilities is broad. This tree, therefore, represents the possible outcomes if one of the options is chosen repeatedly (Fig. 1A). The state space for the task is, however, the product space over the nodes of two of these trees (Fig. 1B), as it is constructed of all combinations of possible outcomes from each individual tree. When the FEV is calculated for one of the options, it is only calculated across the nodes in the full tree that are visited by the optimal policy. This is because the max operator in equation 1 is an expectation over the policy that optimizes choices in each state. Thus, when the FEV is calculated the expectation is taken over the portion of the product space (Fig. 1B) where the expected action value of an option is greater than the other option (thick lines in Fig. 1B). The expectation is not computed over the dotted lines (Fig. 1B) because an optimal policy does not choose these actions. If we examine the distribution of reward probabilities over a representative finite horizon (Fig. 1C) we see that options which have been sampled less have higher expected values, when IEVs are each 0.5. In this example it is less likely that one will encounter a reward probability (q) of 0.5 for options that have been sampled less, and more likely that one will encounter options that have a reward probability greater than 0.8. This increased mass over higher reward probability nodes in the tree drives exploration in bandit tasks.

**Fig 2:**
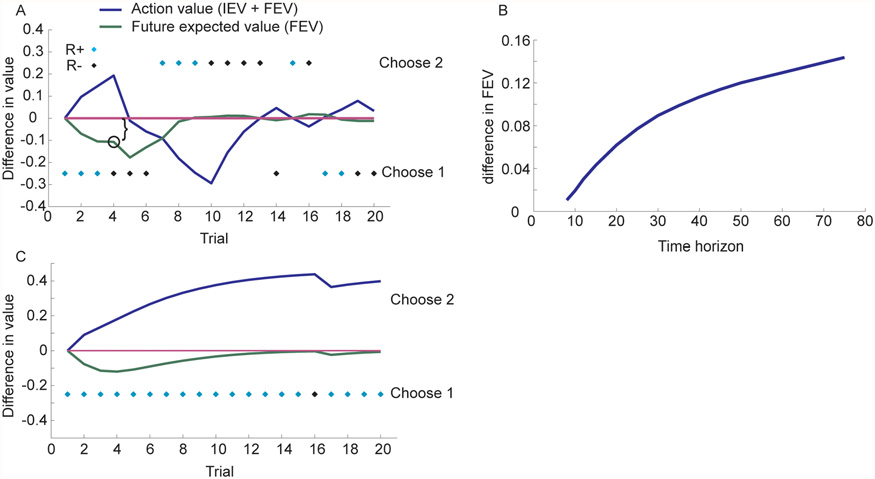
Two armed bandit example. Panels A and C are shown for a 50 trial fixed horizon model. A. Difference in action value between option 1 and option 2. Blue dots indicate rewarded choice, black dots indicate unrewarded choice (R+ = rewarded, R- = unrewarded). Bracket indicates difference in future expected value shown in panel B. Results are for a finite time horizon model with a 50 trial horizon and no discounting. The agent is not following the optimal policy in this example. Choices and outcomes were fixed to illustrate a specific point. B. Difference in future expected value on trial 4 as a function of time horizon. C. Difference in action value in a scenario in which one of the targets is chosen, and it is rewarded every time except in trial 16.

As an example, we examined a scenario in which bandit option 1 was sampled 6 times, and rewarded three times (Fig. 2A). (Note that in this example the agent is not following the optimal policy. Rather we have defined choices and outcomes to illustrate action values under particular scenarios.) The action value for option 1 exceeds the action value for option 2 during the first three trials while it is being rewarded. The FEV, however, of option 2 is larger than the FEV of option 1, even in the first 3 trials, during which option 1 is being rewarded. After option 1 is not rewarded once, it becomes more valuable to sample option 2 (i.e. Q(s,2) > Q(s,1) in trial 5). After option 1 had been sampled 6 times and rewarded three times, its IEV is the same as option 2, which had an expected value of 0.5 because of its prior. However, the action value (IEV + FEV) favors option 2 at this point (i.e. trial 7). If option 2 is then sampled 6 times and rewarded 3 times, the action values of the two options are again the same (i.e. trial 13). The exploration bonus (here taken as the difference in FEV between the two options on trial 4) is also larger when the time horizon is longer (Fig. 2B). This is because option 2 can be exploited for a longer time horizon if it is sampled and found to be better. When the first option chosen is rewarded, and it continues to be chosen and rewarded, the action value of the second option will not exceed the value of the first option (Fig. 2C), given these finite time horizons.

The exploration bonus is driven by three factors. Continuing on the example above, assume option 1 has been sampled and option 2 has not been sampled. First, there is uncertainty about option 2 (i.e. the prior distribution over possible reward probabilities for unsampled options is broad, assuming a vague prior). Therefore, option 2 might be better than option 1. If option 1 cannot be better than option 2, because of the structure of prior knowledge, there is no exploration bonus. The second factor, as shown above (Fig. 2B) is the time horizon [17]. If the time horizon is too short one cannot obtain enough additional rewards when option 2 is found to be better than option 1, to make up for the scenarios (i.e. other episodes of the task) when option 2 is found to be not as good as option 1. This factor relies on the assumption that option 2 might be better than option 1. Third, if option 2 is sampled and it is not as good as option 1, then one can switch back to option 1. On the other hand, if option 2 is better than option 1, then one can stick with option 2. This preference for the option which will be found to be better in the future, drives choices in the present via the max operator over action values in the utility equation (equation 1), which operates on the distribution of future outcomes via the embedded recursion.

### 3-armed bandit novelty task

We next examined a novelty task [5,8,29]. This is a 3-armed bandit task similar in several ways to the 2-armed bandit task described above. The size of the reward is the same for each bandit option, but the probability of receiving a reward when each option is selected differs. In addition to this, however, choice options are replaced by novel choice options at stochastic intervals. Thus, after subjects accumulate experience with the current set of 3 bandit options for a period of time, one of the options is replaced by a novel option. These replacements are stochastic and not known in advance, but they are indicated to the subject. We modeled this task with an infinite horizon, finite state, discounted POMDP. Consistent with the 2-armed bandit, the information state is defined by R_i_,C_i_ for each option. The full information state is now a product space across 3 trees (Fig. 1A), so it is larger.

**Fig 3:**
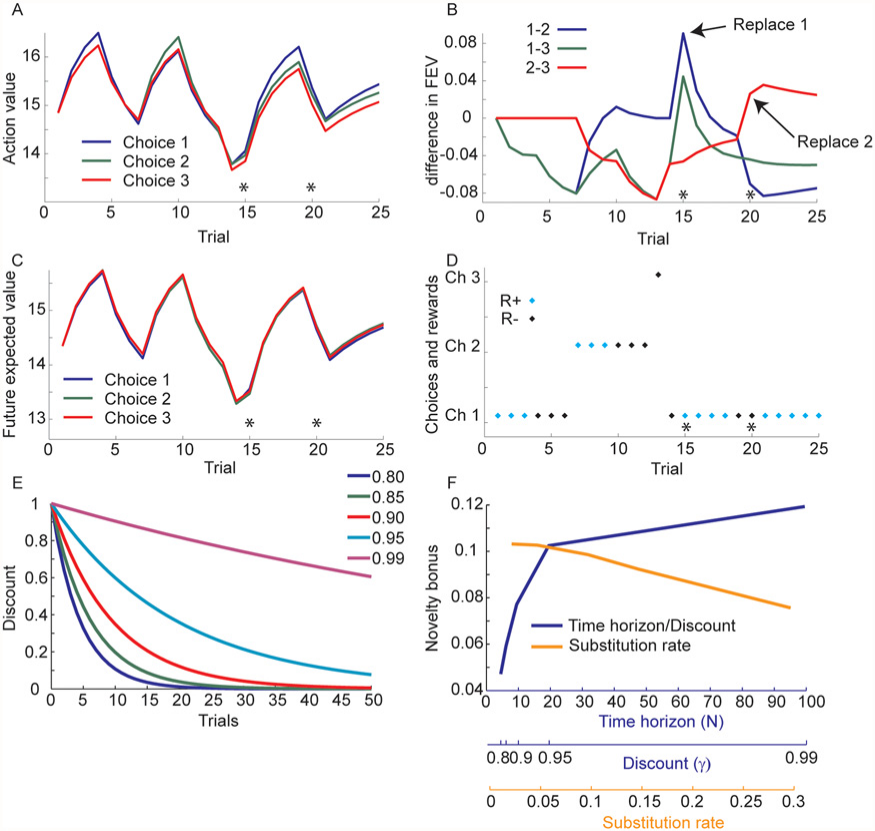
Utilities in the novelty task. Panels A-D, γ = 0.95. A. Total expected value for the three options across a 25 trial sample. Stars indicate trials on which novel choice options were introduced. Colors indicate each choice option. B. Difference in future expected values (FEV: 1–2: difference between future expected value for choosing 1 vs. 2, etc.). C. Future expected values for the three options. Stars indicate trials on which novel choice options were introduced. Colors indicate each of the 3 choice options. D. Choices and rewards for the 25 trial sequence shown in panels A-C. Stars indicate where novel options were introduced. Blue symbols indicate choices that were rewarded (R+), black symbols indicate choices that were not rewarded (R-). Position on the y-axis indicates the choice (e.g. Ch 1 is choose option 1). E. Discount function for different discount rates. F. Exploration bonus (i.e. difference between option 1 and option 2 when option 1 is replaced at trial 15) as a function of discount parameter, and as a function of the probability of substituting a novel option. As discount parameter approaches 1, and the time horizon extends further into the future, the novelty bonus increases. There are 3 x-axes. The first two correspond to plotting the novelty bonus as a function of either the calculated time horizon, or the discount. The x-axis enumerated in trials is the number of trials, N = -1/log_e_(γ), at which the utility is discounted by 1/e. The third is the x-axis for the substitution rate, plotted with γ = 0.95. Substitution rate is p = 0.05 for time horizon line and all other data.

To examine this task we considered a scenario similar to the one examined for the 2-armed stationary bandit. The action value of the chosen option (option 1) increased while it was being rewarded in trials 1–3 (Fig. 3A, for the choices and rewards see Fig. 3D; note that these actions are not chosen by the optimal policy. Rather they were chosen to illustrate the effect of experience with an option). The FEV also increased for all 3 options because of the overall increase in the expected reward in the environment (Fig. 3C). However, similar to what was seen in the 2-armed bandit (Fig. 2A), the FEV was larger for unexplored options (Fig. 3B). Further, when option 1 was replaced, after each of the options had been chosen a few times, its FEV increased relative to the other two options (Fig. 3B, trial 15). Similarly, when option two was replaced on trial 20, its FEV increased (Fig. 3B). As with the 2-armed bandit, when the discount parameter was increased towards 1 (Fig. 3E), the exploration bonus increased (Fig. 3F). Thus, when a long time-horizon is available to exploit a novel option if it is found to be more valuable, the FEV for exploring that option increases. Every time a novel option is introduced, it is equivalent to resetting that option to the beta(1,1) prior, resetting it to the start of the tree (Fig. 1A). Thus, uncertainty drives an exploration bonus as long as a sufficient time horizon is available to exploit the novel option if it turns out to be better than the alternative options available. Correspondingly, the substitution rate of novel options also affects the novelty bonus, by effectively limiting the time horizon (Fig. 3F). If the substitution rate is high, one likely will have less time to exploit novel options that turn out to be good, before they are again replaced.

### Exploration in non-stationary bandits

**Fig 4:**
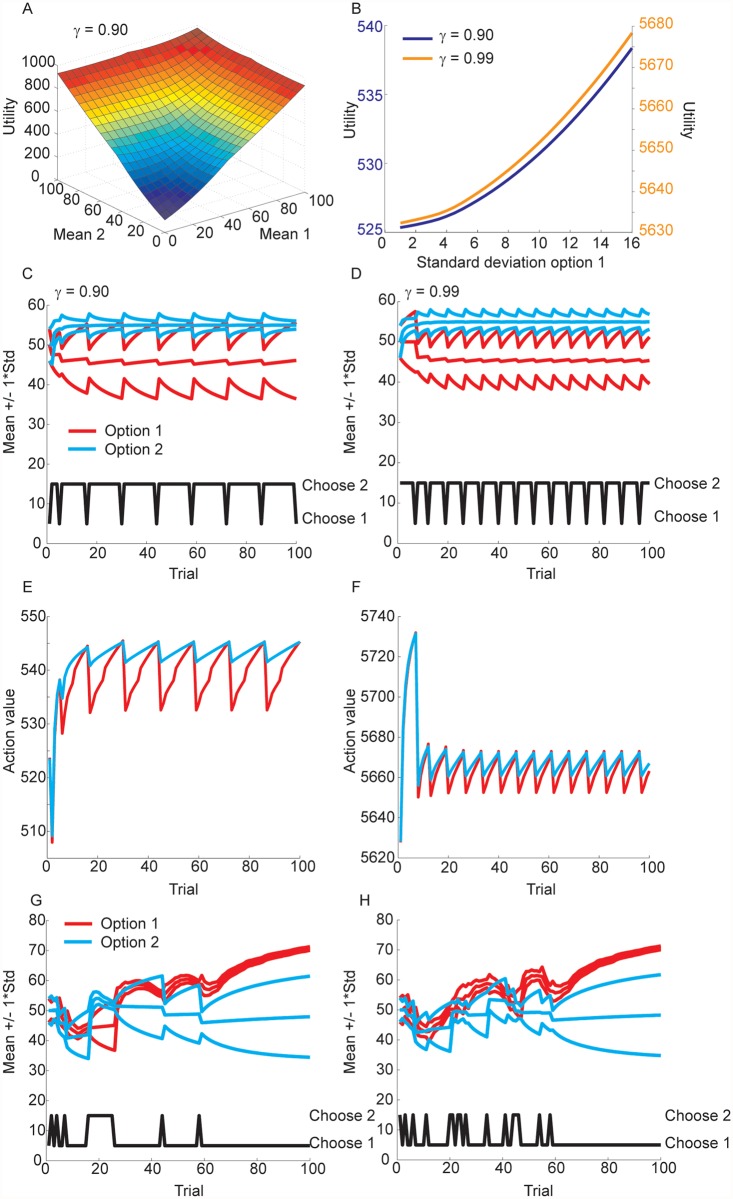
Utilities in nonstationary 2-armed bandit. A. Utility as a function of mean of option 1 and mean of option 2, with standard deviation of both options set to 4 and discount rate, γ = 0.90. B. Utility as a function of standard deviation for 2 discount values when mean is 50 for both options and standard deviation is 4 for option 2. C. Estimate of mean and standard deviation of options 1 and 2 as they are sampled under a condition where means are fixed at 45 and 55. Black line indicates choice of option 1 (y = 5) or option 2 (y = 15). Discount rate γ = 0.90. D. Same as panel C, except γ = 0.99. E. Plot of action value for two options for data plotted in C, γ = 0.90. F. Action value for two options for data plotted in panel D, γ = 0.99. G. Example sequence of samples and estimates of mean and variance, γ = 0.90 for means drawn from the generative model. H. Example sequence of samples and estimates of mean and variance, γ = 0.99.

To examine exploration in related bandit tasks, we used an infinite horizon, discounted, continuous state, POMDP to model a non-stationary two-armed bandit task [9]. The information state in this model is given by the mean and variance of the bandits, which are the sufficient statistics for the two processes. The bandits in this task returned continuous valued rewards (e.g. 0–100). The means of the returned values for each bandit were non-stationary in time, following independent, random walks that decayed to 50. The actual reward earned on an individual trial was given by a sample from a Gaussian distribution with the current mean, and a standard deviation of 4. The IEV is given by the estimated mean of each bandit. The utility depends on the estimated means of the two options (Fig. 4A) as well as the estimated variance of the options (Fig. 4B). The effect of variance on utility also depends on the time-horizon (Fig. 4B). The variance has a larger effect when the time horizon is longer. The effect of the variance of the utility can be understood in the framework developed above for the stationary bandit (Fig. 1). Specifically, when an option is not sampled its variance grows because of the nonstationarity of the underlying generative model, effectively driving it backwards in the tree (Fig. 1A). On the other hand, when an option is sampled its variance decreases, effectively driving it forwards in the tree (Fig. 1A). Thus, an option which has not been sampled for several trials becomes similar to a novel option, and it should be explored.

We examined the choice sequence of the algorithm for some examples. If we consider an artificial case where the means are locked at 45 and 55 (but the algorithm still assumes the means are non-stationary), and compare the sampling under two different discount parameters (effective time horizons) we see that the algorithm periodically samples the option with a smaller estimated mean, as its variance grows (Fig. 4C). In addition, when the discount parameter is larger (γ = 0.90 vs γ = 0.99) the algorithm samples more often, consistent with the larger difference in utility for a given standard deviation for larger discount parameters (Fig. 4B). This can also be seen clearly in the action values (Fig. 4E and F—Note that the algorithm stochastically sampled option 1 first in panel E and option 2 first in panel F, which gives rise to the initial downward vs. upward fluctuation). With the means fixed, the action values depend only on the variance of the two processes, if we ignore the decay of the process to 50. When an option is sampled its variance decreases and its utility decreases, and when an option is not sampled its variance increases and its utility increases. The combination of these eventually drives the action value of the recently unsampled option to exceed the action value of the option currently being sampled (Fig. 4E and F), and the option which has not been recently sampled is then sampled. This can be seen in example sequences drawn from the actual generative process as well (Fig. 4G-H, the actual process values are identical for these two examples). In this case when the algorithm is modeled with a longer time horizon it samples more (Fig. 4H).

### Information sampling

**Fig 5:**
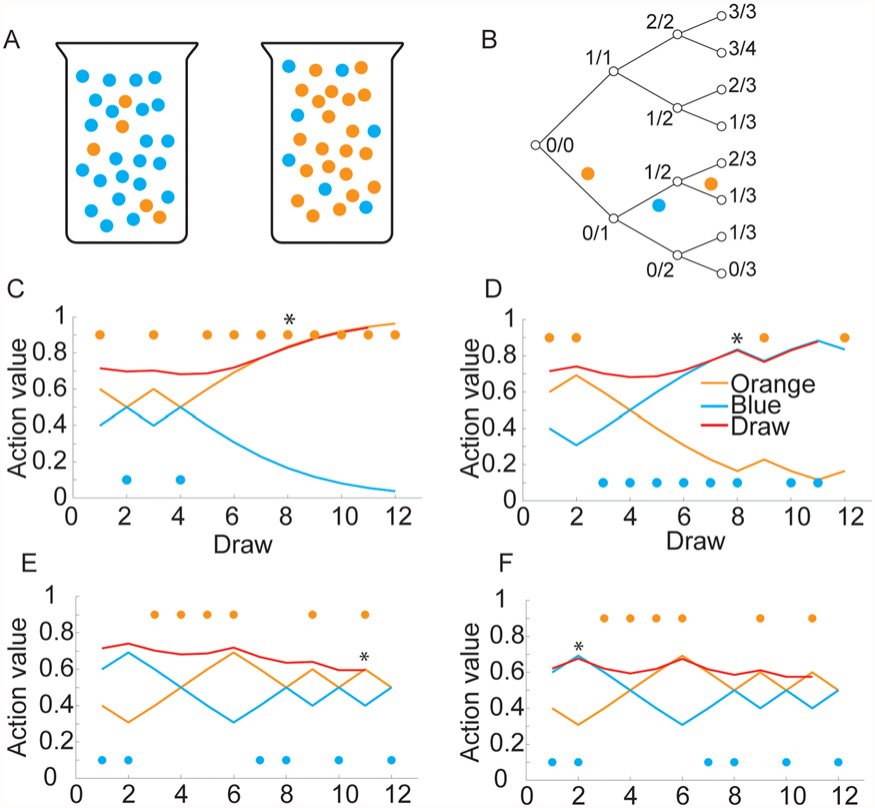
Beads task. A. Example distribution of beads in the beads in the jar task. B. State space for beads task. Example sequence of draws is taken from panel C. C-F. Action value for the three choice options as a function of draws for example sequences. Bead outcomes are shown as orange and blue beads. The star indicates the first trial on which the expected value of choosing an urn is greater than the expected value of drawing again. In this case an ideal observer would guess the urn with the highest value. Not that this is the value after seeing the bead shown in the corresponding trial. In panels C-E, cost to sample is C(s_t_,a) = -0.005. In panel F, C(s_t_,a) = -0.025.

We next examined an information sampling task, often referred to as the beads or urn task [4,5,20,21,30]. In this task subjects are shown a sequence of beads drawn from one of two possible urns (Fig. 5A). One of the urns has q orange beads and 1-q blue beads and the other has q blue beads and 1-q orange beads. After each bead is drawn subjects have three choices. They can either draw another bead from the urn, guess that beads are being drawn from the predominantly blue urn, or guess that beads are being drawn from the predominantly orange urn. Sampling another bead usually involves an explicit cost-to-sample. In other words, subjects are charged for collecting more information. In this task, the value of choosing an urn is given by the IEV, because no more samples are allowed after an urn is chosen so FEV is zero, whereas the value of sampling another bead is given by the FEV (minus the cost-to-sample), because there is no reward if one does not try to infer the urn. Thus, this task explicitly sets up a trade-off between immediate and future expected rewards, and in this sense it is similar to the explore-exploit trade-off in bandit tasks.

In most cases subjects are told that they can draw only up to a maximum number of beads and after the last bead is drawn they have to guess an urn. As such, the task can be modeled as a finite horizon, finite state, undiscounted, POMDP. The information state space is simpler than the state space in the bandit task, as it is given by a single tree (Fig. 5B), where instead of rewards and no rewards, the state is given by the number of blue (or orange) beads that have been drawn, and the total number of beads drawn. These form the sufficient statistics for the process. As one draws beads, one works through the state space, similar to the situation with the bandit tasks. For example, the first 3 bead draws for the example sequence shown in Fig. 5C would go through the set of states shown (Fig. 5B). Unlike the bandit task, this task was modeled with an uninformative prior on bead draws, because it is normally implemented by showing subjects one bead before asking them to decide [21]. The action values for guessing either of the urns or sampling again show that the value of guessing an urn increases as evidence for that urn increases (i.e. more beads drawn of the corresponding color), and decreases as evidence for the urn decreases, in a cumulative fashion (Fig. 5C-F). The value of sampling again is initially above the value of guessing an urn, but at some point it drops slightly below. Note that without a cost-to-sample (C(s_t_,a) = -0.005 in panels C-E and C(s_t_,a) = -0.025 in panel F) it is always best to sample all of the available beads. To examine the effect of the cost-to-sample, we calculated values for two costs, on identical sequences of bead draws (Fig. 5E-F). When the cost was lower (C(s_t_,a) = -0.005; Fig. 5E), it was optimal to delay the decision until after the 11^th^ bead was drawn, whereas when the cost was higher (C(s_t_,a) = -0.025; Fig. 5F) it was optimal to decide after 2 beads. This task can be considered a pure exploration task: how long does one explore before committing to (exploiting) one of the choices? This is similar to exploring a novel option for several trials, while always considering whether to switch back to the known option, or sticking with the novel option. As the certainty about which urn is being drawn from increases, picking an urn (which will deliver an IEV), as opposed to drawing again (which is valuable because of the FEV), becomes more valuable.

### Foraging tasks

**Fig 6:**
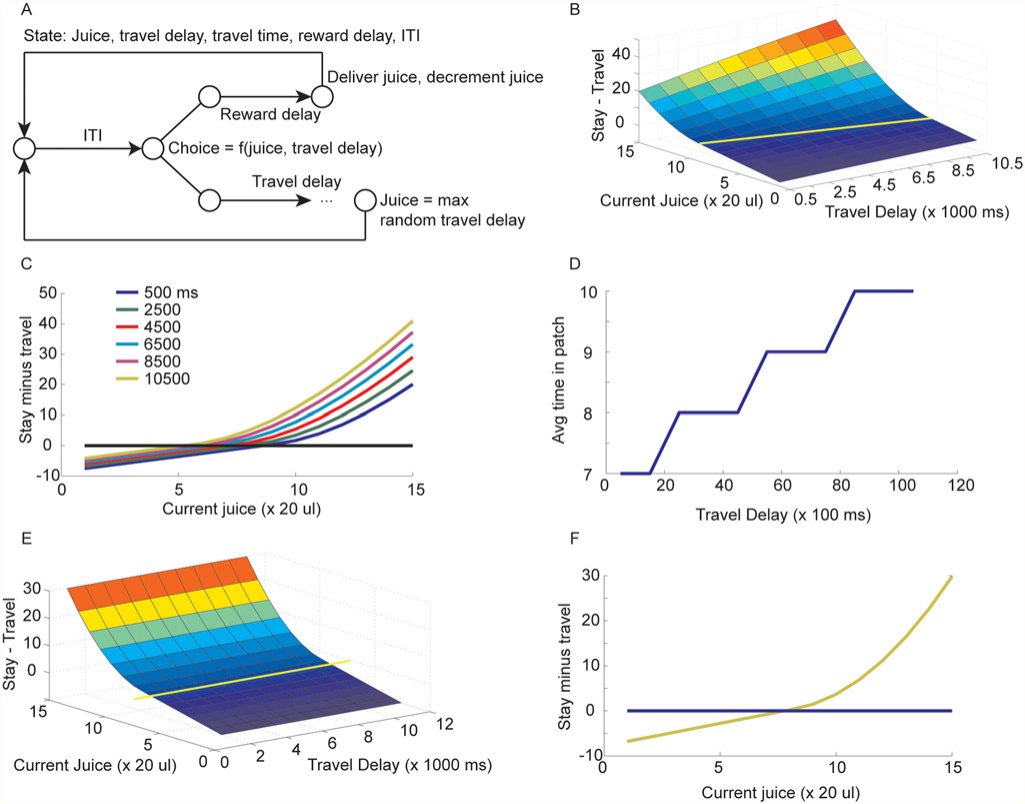
Patch leaving foraging task. A. State space model for the task. The full state space has been collapsed. The state space shown would be repeated, one for each juice, travel delay combination. We show this here by indexing the choice state by these variables. B. Difference in action value for stay in patch vs. travel as a function of current juice and current travel delay. Yellow line indicates point of indifference between stay and travel. C. Difference in action value (same data as plotted in B) with each line representing a different travel delay. D. Average time in patch as a function of current travel delay. Note the curve is discontinuous because of the discretization of the problem. E. Difference in utility with an infinite, undiscounted time horizon. Yellow line indicates point of indifference between stay and travel. F. Difference in action values with an undiscounted, infinite time horizon. Note that the travel delay does not affect value, as would be expected.

**Fig 7:**
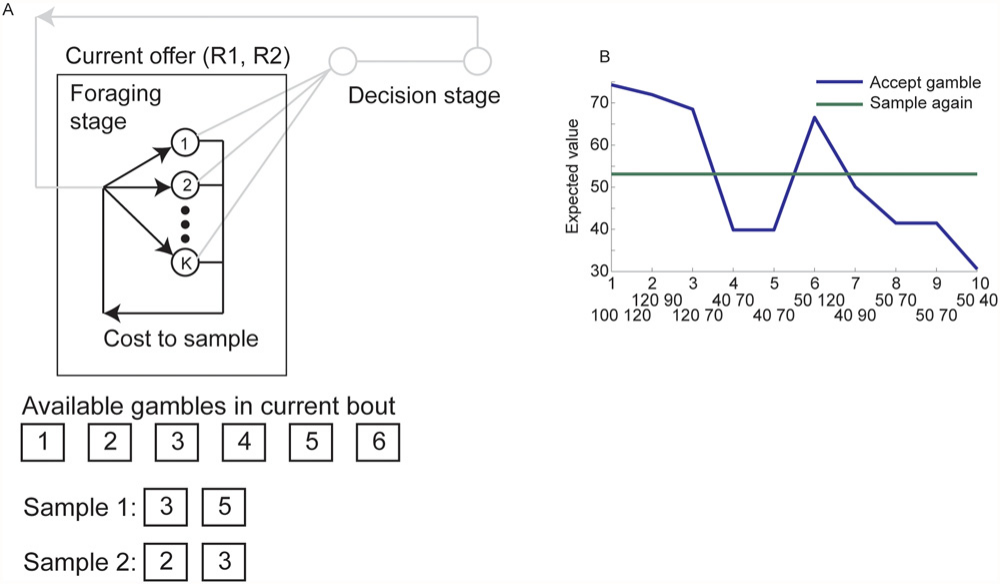
Sampling foraging task. A. State space model for the foraging task. The numbers in the circles indicate one of the offer pairs. As there were 6 available individual gambles, there were 15 offer pairs possible in each foraging round. Bottom of panel shows gambles that would be available in a specific foraging bout. In each trial subjects are shown a randomly sampled pair from the 6. If they accept the pair, they move on to the decision stage. If they sample again, a new pair is shown, and they have to decide whether to accept the pair, or sample again, etc. B. Expected value for accepting the current gamble or sampling again for an example sequence of draws. Option below trial number is the option pair that was presented on that trial.

The final tasks we considered were foraging tasks. Much like the tasks examined above, these tasks trade-off immediate and future expected values. Should one stay in the current patch whose resources are being depleted (i.e. choose IEV) or travel to a new patch (i.e. choose FEV) [19]? Or, should one sample again (i.e. choose FEV) or commit to the current gamble on offer (i.e. choose IEV) [18]? The state spaces for these tasks differ in a fundamental way from the state spaces in the bandit and information sampling tasks (Fig. 6A and 7A). The state spaces for the foraging tasks are recursive. Stated another way, the state spaces for the foraging tasks do not represent learning or information accumulation. Learning or information accumulation are not recursive because you do not return to the same state (technically, this is not completely accurate, as one can with some probability, return to a previous state in either the non-stationary bandit or the novelty bandit). Rather, in the foraging tasks the current state is provided to the animal and the animal does not have to estimate beliefs or distributions over states. Therefore these tasks are MDPs, as opposed to POMDPs where the state is hidden. In the foraging tasks one observes the state directly.

### Patch leaving task

In the patch leaving time task the subjects chose between staying in the current patch or traveling to a new patch [19] in each trial. The state relevant to choices is defined by the current amount of juice and the travel delay. If they stay in the current patch, they receive a (slightly delayed) reward, and the amount of reward that they will receive in the next trial if they again choose to stay in the current patch is decreased. If they choose to leave the current patch they have to wait for a known travel delay and they receive no immediate reward. The amount of reward that will be received in the new patch is reset to a fixed level and the travel time to the next new patch is sampled from the distribution of possible travel times (Fig. 6A). The patch leaving time task was modeled as an infinite horizon, discounted MDP. The relevant state variables when a decision is made are given by the current travel delay and the current reward estimate (Fig. 6A). From the model one can calculate the difference in action value for staying in the patch vs. leaving for another patch (Fig. 6B and 6C). It can be seen that the longer the travel time, the longer one stays in the patch (Fig. 6D), consistent with what was shown previously with heuristic models [19]. However, this effect only occurs for discount parameters less than 1. The undiscounted model is insensitive to finite travel times (Fig. 6E and 6F). This is because undiscounted infinite horizon MDPs are insensitive to finite time delays. Stated another way, if K is the mean first passage time to a state s_t_ = j and from state j one follows the optimal policy, then with an infinite horizon the value function can be written [26]:

$$v_{N}^{\pi }\left(s\right)\;=\;\underset{N=\infty }{\lim}\frac{1}{N}{\left\langle \sum_{t\;=\;1}^{K-1}r\left(s_{t},a\right)\right\rangle }_{s}^{\pi }+\underset{N=\infty }{\lim}\frac{1}{N}{\left\langle \sum_{t\;=\;K}^{N-1}r\left(s_{t},a\right)\right\rangle }_{s}^{\pi }$$

From this it can be seen that actions taken prior to entering state j, at time K, do not matter. This is because the first sum is finite if the rewards are finite, and so it goes to zero in the limit. In the foraging task, if K is the time to get to the inter-trial interval (ITI) after choosing to travel, it doesn’t matter how long K is for finite K.

### Foraging-by-sampling

The final task is a variant on standard foraging tasks. The state for this task is given by the current gamble pair on offer and the state space includes all the possible gamble pairs. In most foraging tasks a decreasing marginal reward in the current patch eventually drives the action value to leave the patch above the action value to stay in the patch, because leaving has a fixed expected value. This task, however, used a paradigm in which one samples, in each trial, two gambles from a set of six possible individual gambles (Fig. 7A). The six individual gambles from which the pairs were drawn were shown for the current foraging bout and their reward values were known (e.g. gamble 1 may have had a value of 12 points). In each round, a pair of gambles from the set of individual gambles was sampled (15 possible pairs assuming sampling without replacement from the 6, and symmetry of gambles). For example, if the gambles for a given session were g1 … g6, a subject might be shown in a single trial g3 and g5. They then have to decide whether to engage with that offer pair, or sample again. If they sampled again, a new pair was drawn from the current set of six possible gambles (perhaps g2 and g3). Every time the subjects sampled again they also incurred a cost-to-sample. (Note that a cost-to-sample is paid at the time of sampling, and it does not decrease the value of future gambles, in an MDP.) If they decided to accept the offer, they moved to a decision stage. In this stage the probability that the reward associated with each gamble would be delivered was revealed, and this probability was randomly assigned to each gamble every time the decision stage was entered. The subjects had to choose one of the two gambles in the decision stage based on its magnitude and the associated probability. For example they might be choosing between p_1_g_1_ and p_4_g_4_ where pi is the probability that the subjects will receive reward gi if they choose that gamble in the decision stage. The agent then selects the gamble that has the highest expected value.

The value of sampling again is given by the FEV. The FEV is not equal to the average values of the individual gambles. The FEV is the expected value of future draws (see methods), plus the cost-to-sample. The time horizon is long, and many future samples could be drawn. However, the cost-to-sample decreases the value of future samples linearly with time, when viewed from the present decision. This can be compared to exponential discounting which exponentially decreases the value of future samples. With a sufficient time horizon the FEV is fixed. The task provided no explicit time horizon so we modeled it as a finite (although long) time horizon MDP. Therefore one simply samples until the IEV of the offered pair exceeds the (constant) FEV (Fig. 7B). It is important to point out that sampling more in this foraging task, unlike the beads task, does not improve the IEV. In other words, the IEV does not necessarily increase with samples, although one can sample a pair with a better IEV. This is related to the state space of the problem. Additionally, without a cost-to-sample, the optimum strategy would be to sample until the pair with the highest value is drawn. The cost-to-sample creates a situation where choice of a gamble pair that is not the largest is optimal, because it may cost too much to obtain a better pair.

## Discussion

We have applied markov decision process models (MDPs/POMDPs) to choice tasks that have been used to study the explore-exploit tradeoff, information sampling and foraging. The models allowed us to determine the normative choice mechanisms in these tasks, and therefore they provide insight into their similarities and differences. All of the tasks manipulate trade-offs between immediate and future expected values (IEVs vs. FEVs). Temporal-discounting tasks explicitly manipulate this trade-off. We have not considered them in the present study, but we have modeled them previously using MDPs [5]. Normative exploration in the bandit tasks can be defined as the choice of an option whose IEV is smaller but whose FEV is larger than an option with which one has more experience. This also drives exploration when there is non-stationarity, or when novel options are presented, because both of these increase uncertainty. In information sampling tasks an explicit trade-off is setup between sampling again, the action value for which is driven by the FEV (plus a small cost-to-sample), vs. inferring an option, the action value for which is driven by the IEV. Finally, foraging tasks also present the option of staying in the current patch, which has a larger IEV but a decreasing FEV, or traveling to a new patch, an action for which the IEV is zero but the FEV is larger. Across these tasks the IEVs and FEVs are calculated in different ways. In other words, the mechanisms that underlie value estimation differ across tasks. When trying to understand the neural circuits and underlying neural processes that carry out decisions in these tasks, it will be important to understand how these computations are carried out in the brain.

### Task specific results

We began by examining the explore exploit trade-off in a two-armed bandit task, in which the reward amount for both options was the same, but they differed on the fraction of times that they were rewarded. Bandit tasks have been used to study learning in healthy and clinical populations [31,32]. In the first few trials there is value to sampling both options, and unsampled options have a larger FEV. This future expected value depends on three factors. First, the distribution over possible reward probabilities for the unsampled option is broad, given by the prior. Thus, the unsampled option may be more rewarding than the options which have been sampled. Second, if the unsampled option is sampled, and it is not as good as the other options, the subject can switch back to the other options. However, if the (previously) unsampled option is better than the other options, the subject can stick with it. Finally, the time horizon must be long enough to reap the rewards of investing samples in the novel option.

Heuristically, one could consider the following approximate example. Assume that one has sampled one of two available options (call it option 1) and found that it is being rewarded 70% of the time, and that one now has 100 more trials. One could then sample the alternative option (option 2) 10 times. If option 2 is rewarded 80% of the time one could then stick with that option, gaining on average 80 rewards over the 100 trial horizon. If it is found that option 2 is only rewarded 20% of the time, then one could switch back to option 1, gaining 0.2*10 + 0.7*90 = 65 rewards on average. If option 2’s (i.e. all option 2’s that one encounters, in repeated plays of the task) are either rewarded 80% of the time, or 20% of the time, the average reward with this simplified strategy will be 72.5 over the 100 trials, whereas it would only be 70 if one always stuck with option 1. The 2.5 additional rewards on average is the exploration bonus. It depends on the possibility that the novel option is better than the current option, the fact that one will switch back to the alternative if it is better than the novel option, and having a sufficient time horizon.

We also examined two other tasks which are extensions of the bandit task. Specifically, a non-stationary bandit [9], and a novelty task [5,8,29,33]. In the non-stationary bandit the mean reward magnitudes of the two options follow independent random walks. When an option is sampled several times, a relatively accurate (i.e. low variance) estimate of its mean can be derived. However, when an option is not sampled, the distribution of its mean becomes broad. When a random walk is not observed for a period of time, the variance of its estimate grows linearly with time. One way to conceptualize this, relative to the stationary bandit, is to say that when an option has not been sampled for some time, it becomes like a new option, and there is value in exploring it. This is true of any tasks that have an underlying non-stationarity in the reward [34]. It is, however, variance in the estimate of the mean that drives the exploration bonus. When the variance gets large, the option might be better than the current options, and exploration is advantageous. Similarly in the novelty task, when a novel option is substituted for one of the options that has been sampled, the reward probability for the novel option is unknown, and therefore it is valuable to explore it.

Next we examined the beads or urn task [4,5,20–22,30,35]. This is an information sampling task, similar in structure to other sampling tasks [36]. The POMDP model for this task only optimized choices in single trials with an explicit cost-to-sample. It did not optimize reward rates over multiple trials. Subjects are given the option to sample as much information as they would like, before guessing an urn. The choice to sample rests on the belief that the FEV of sampling is greater than the IEV of guessing an urn. In this respect, sampling is similar to exploring, as it is a choice in favor of the FEV, relative to the IEV. It differs from exploration, however, in that exploration in bandit tasks usually has some IEV. That is, choice of the unknown option in bandit tasks usually leads to some reward. This does not need to be true in general. In information sampling tasks, however, choosing to sample usually leads to zero IEV (or a slightly negative IEV, given by the cost to sample). In this way, sampling is more similar to foraging. It is also worth pointing out that reaction time versions of perceptual inference tasks can be modeled within a framework that is equivalent to the approach used here to model information sampling [37,38]. Perceptual inference tasks, as well as many other choice tasks, are often modeled using a drift-diffusion framework, and it is assumed that when an evidence bearing particle crosses a threshold a decision is made. The “threshold” crossing is a choice to stop sampling. It is often inferred for drift diffusion models in perceptual inference tasks, on the basis of behavioral reaction times. But with an MDP the threshold can be calculated dynamically, on the basis of current levels of belief, costs-to-sample, and transition probabilities [38]. Thus, an optimal threshold can be inferred for any tractable task.

The final tasks we considered were foraging tasks. These tasks also trade-off immediate vs. future expected values. The choice to forage leads to a zero IEV. The action value of choosing to forage is entirely an FEV. Foraging tasks differ from the tasks considered above, because their state spaces have a recursive structure and the state is observed, not inferred from information bearing observations. The tasks loop through their recursive state spaces over and over again. The choice is defined as a comparison between current and future stochastic offers. The current offer can be to stay in the patch and collect an approximately known, decreasing reward, or take the current pair of gambles that have been offered. The future stochastic offer can be explicitly calculated from the information given. It is either the value of a new patch, given the current travel time, or the expected value of the decision stage for the set of gambles that can be drawn from the current set. These average values are fixed with a sufficient time horizon. Therefore the strategy is to either stay in the current patch until the reward value drops below the value of leaving, or to sample gambles until the sampled gamble is worth more than the expected value of future samples. In foraging tasks there is generally no updating of distribution estimates, and therefore foraging differs fundamentally from exploration, in this respect.

### Factors driving choice strategies across tasks

There are two important factors that drive choice preferences across these tasks. The first is uncertainty, and the second is the time horizon. Uncertainty affects these models in two ways. First, in bandit tasks when novel options are available, or equivalently when non-stationary options have not been explored for some time, the distribution of possible reward values is broad and uncertainty is high. Therefore, sampling the options a few times to learn about them is valuable, given a sufficient time horizon. The value of this uncertainty is driven by the future expected value. The sampling itself, however, decreases uncertainty about the options. When one learns that an option either is, or is not valuable, then one can act accordingly. Thus, increased uncertainty drives value through the FEV. Because we used models that maximize expected reward, uncertainty does not affect IEV. However, as uncertainty can lead to a larger FEV, decreasing uncertainty and therefore decreasing FEV increases the relative importance of IEV on the total action value. The same reasoning applies to the information sampling tasks. As long as uncertainty is high, the FEV is high. When uncertainty is decreased, however, the IEV of guessing an urn becomes larger. Interestingly, and in contrast to this, increasing uncertainty in temporal-discounting tasks actually decreases preference for delayed, larger rewards [5]. (Temporal-discounting tasks are tasks in which subjects are offered a choice between an immediate smaller reward and a delayed larger reward.) This is because of the state space of temporal-discounting tasks. One can model temporal discounting tasks using an MDP which, at each time step, includes the possibility of exiting the path to the reward and terminating in a state with no reward, with some probability. If this probability of terminating in a no reward state increases, it becomes less likely that one will get to the reward, for a fixed delay to the reward. Interestingly, this is thought to be a fundamental factor that drives crime [39].

**Fig 8:**
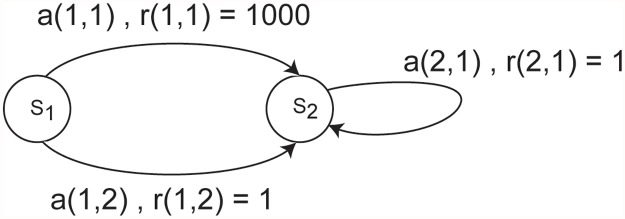
Example MDP. Note that from state 1, picking action 1 leads to a reward of 1000, and a deterministic transition to state 2. Picking action 2 from state 1 leads to a reward of 1 and a deterministic transition to state 2. Only one action is available in state 2. It leads to a reward of 1 and a deterministic self-transition.

Time horizon is also important. In infinite horizon problems the time horizon is controlled by the discount parameter. In bandit problems, the time horizon affects the relative value of exploration. In stationary problems the time-horizon affects the relative value of exploring novel or unknown options. Longer time-horizons, or discount parameters closer to 1, increase the value of exploration. In non-stationary environments this relationship is more complex, as the non-stationarity limits the effective time-horizon of any policy. In foraging tasks, however, time horizon is also important. In undiscounted infinite horizon problems, travel times are irrelevant. If one has an infinite, undiscounted time horizon, then any finite travel time does not affect value. In the non-stationary bandit task, when the discount parameter approaches one, the algorithm samples options with lower means more often. As another example, consider the simplified MDP shown in Fig. 8. The undiscounted, infinite horizon solution to this problem does not favor action 1 over action 2 [40] because the relative value of this initial transient reward will be zero in the infinite time limit. Methods such as sensitive discount optimality exist to deal with such situations, although these can only be applied to tractable state spaces [40]. However, a discounted MDP favors action 1, in this case. This suggests that temporal discounting, in some form, is ubiquitous because it is always biologically (or computationally) relevant. Whether discounting is specifically exponential or hyperbolic, or takes on some other form is less the issue. More important is some sort of monotonic decrease in the value of future rewards with distance into the future.

### Conclusion

The explore-exploit trade-off is often modeled with heuristics. A strong criticism of heuristics is that they explain no more than they assume, and tell us no more than the data does [28]. Heuristics, however, can provide reasonable solutions to engineering problems, often provide insight into patterns in the data, and may better approximate behavior than normative models [15]. For example, recent work has explicitly examined the role of noisy vs. directed exploration, and found that human subjects use both directed and noisy exploration strategies [31]. In some cases, however, heuristics can be difficult to interpret. For example, the beta or inverse temperature parameter in delta-rule reinforcement learning (DRRL) is often thought to control the “explore-exploit” trade-off. This parameter can only control noise in choice processes, however, and standard implementations of DRRL do not turn this noise down as reward values are learned. Therefore, exploration cannot be differentiated from noise in the choice process using this parameter and poor learning looks like exploration. Several more sophisticated variants including Thompson sampling [41,42] and related algorithms [43], however, decrease exploration with learning and can achieve minimal regret.

In an MDP framework exploration need not be undirected or noisy. Exploration can be an intentional, directed, normative strategy if there is sufficient knowledge of the environment and the agent has sufficient computational resources. One does not necessarily explore so much as one learns or accumulates information (bandit or information sampling tasks) until the additional information indicates that an alternative choice is better. Every choice delivers some information, because one is always transitioning through states as choices deliver information in these tasks. Equivalently, leaving the current patch in a foraging task is an explicit calculation of the relative value of traveling to a new patch, the expected value of which is characterized by some probability distribution over patch values. It is possible that animals have relatively unsophisticated strategies for dealing with these issues. It seems likely, however, that they have developed at least a good approximation to the underlying normative utilities, at least in tasks that match the animal’s ecological nitch or on which the animals have extensive experience.

## Methods

### Markov decision processes

We modeled the tasks using markov decision processes with either observable (MDP) or partially observable (POMDP) states. Tasks were modeled as finite or infinite horizon, discrete time, and discounted (i.e. with a discount parameter *γ* < 1) or undiscounted (i.e. with a discount parameter *γ* = 1) as indicated in the manuscript. Some models also included a cost-to-sample. For discrete state models the utility, *u*, of a state, *s*, at time t is

$$u_{t}\left(s_{t}\right)\;=\;\underset{a\in A_{s_{t}}}{\max}\left\{r\left(s_{t},\text{a}\right)+C\left(s_{t},a\right)+\gamma \underset{j\in S}{\sum }p{(}_{j}|s_{t},a)u_{t+1}\left(j\right)\right\}$$

where $A_{s_{t}}$ is the set of available actions in state s at time t, *r*(*s*_*t*_,*α*) is the reward that will be obtained in state s at time t if action a is taken. The variable *C*(*s*_*t*_,*α*) is a cost-to-sample, which may be zero. The summation on j is taken over the set of possible subsequent states, S at time t+1. It is the expected future utility, taken across the transition probability distribution *p*(*j*|*s*_*t*_,*α*). The transition probability is the probability of transitioning into each state j from the current state, s_t_ if one takes action a. The *γ* term represents a discount factor. The terms inside the curly brackets are the action value, *Q*(*s*_*t*_,*α*) = *r*(*s*_*t*_,*α*) + *C*(*s*_*t*_,*α*) + *γ*Σ_*j*∈*s*_ *p*(*j*|*s*_*t*_,*α*)*u*_*t*+1_(*j*), for each available action. For continuous state models the utility is

$$u_{t}\left(s_{t}\right)\;=\;\underset{a\in A_{s_{t}}}{\max}\left\{r\left(s_{t},a\right)+C(s_{t},a)+\gamma \int_{S}^{}p(j|s_{t},a)u_{t+1}(j)dj\right\}$$

All state integrals over continuous states were calculated with discrete approximations. Equations 1 and 2 assume a reward maximizing agent, through the max operator.

For discrete state, finite horizon models with tractable state spaces, we used the backward induction algorithm to calculate utilities and action values [28]. This was done for the 2-armed stationary bandit, beads and sampling foraging tasks. With a finite horizon the final state delivers a reward, but no further actions are possible. Therefore, if we start by defining the utilities of the final states, we can work backwards and define the utilities of all previous states. Specifically, the algorithm proceeds as follows [40], where N is the final state.

1. Set t = N

$$u_{N}\left(s_{N}\right)=r\left(s_{N}\right)\;for\;all\;s_{N}\;\epsilon \;N.$$

2. Substitute t-1 for t and compute

$$u_{t}\left(s_{t}\right)\;=\;\underset{a\in A_{s_{t}}}{max}\left\{r\left(s_{t},a\right)+C(s_{t},a)\;+\gamma \sum_{j\in S}p(j|s_{t},a)u_{t+1}(j)\right\}$$

Set

$$A_{s_{t},t}^{*}\;=\;arg\underset{a\in A_{s_{t}}}{\max}\left\{r\left(s_{t},a\right)+C(s_{t},a)\;+\gamma \sum_{j\in S}p(j|s_{t},a)u_{t+1}(j)\right\}$$

3. If t = 1 stop, otherwise return to 2.

The non-stationary 2-armed bandit, novelty and patch-leaving foraging tasks were modeled as infinite horizon POMDPs or MDPs. The utilities were fit using the value iteration algorithm [40]. This algorithm proceeds as follows. First, the vector of utilities across states, *v* ^0^, was initialized to random values. We set the iteration index, n = 0. Then computed:

$$v^{n+1}\;=\;\underset{a\in A_{s_{t}}}{\max}\left\{r\left(s,a\right)+\gamma \underset{j\in S}{\sum }p(j|s_{t},a)v^{n}\left(j\right)\right\}.$$ (3)

After each iteration we calculated the change in the value estimate, $\Delta v\;=\;v^{n+1}-v^{n}$, and examined either ||Δ*v*||<∈ or span(Δ*v*) <∈. The span is defined as.*span*(*v*) = *max*_*s∈S*_ *v*(*s*)—*min*_*s∈S*_ *v*(*s*) For infinite horizon undiscounted models the value continues to grow with iterations of equation 3, but the spans converge [40]. This is because the final state values are the average costs per stage plus a differential. This only applied to the foraging patch leaving example with discount parameter equal to 1. We only examined differential values, in that case, so the average cost per stage is subtracted out, because it is added to all states.

We also used approximate methods for the non-stationary 2-armed bandit and the novelty task, as their state spaces were intractable over relevant time horizons. For these POMDPs we defined a basis, and then approximated the utility with

$$\hat{v}\left(s\right)=\sum_{i=1}^{M}a_{i}\phi_{i}\left(s\right).$$ (4)

In all cases we used fixed basis functions so we could calculate the basis coefficients, a_i_ using least squares techniques. We assembled a matrix Φ_*i*,*j*_ = ϕ_*i*_(*s*_*j*_), which contains the values of the basis functions for specific states, *s*_*j*_. We then calculated a projection matrix

$$H\;=\;{\Phi (\Phi^{'}\Phi )}^{-1}\Phi '$$ (5)

And calculated the approximation

$$\hat{v}\;=\;Hv.$$ (6)

The bold indicates the vector over states, or the sampled states at which we computed the approximation. When using the approximation in the value iteration algorithm, we first compute the approximation,$\;\hat{v}$. We then plug the approximation into the right hand side of equation 3, $v^{n+1}\;=\;\underset{a\in A_{s_{t}}}{\max}\left\{r(s,a)+\gamma \sum_{j\in S}p(j|s_{t},a){\hat{v}}^{n}(j)\right\}$. We then calculate approximations to the new values ${\hat{v}}^{n+1}\;=\;Hv^{n+1}$. This is repeated until convergence.

For basis functions we used piece-wise polynomials and/or b-splines [44]. For b-splines see [44]. For piecewise polynomials, the first basis functions are given by *h*_*i*_(*x*) = x^*i-1*^. For an order K spline (i.e. for cubic K = 3), i goes from 1 to K+1. In addition to these global polynomials, we also add *hj*(*x*) = (*x-t*_*j*_)^*K*^ for the J knots, *t*_*j*_. Because all of the state spaces were multidimensional and the piece-wise polynomial basis varied between knots, we also had to compute products of the basis functions across dimensions. Computing the full tensor product basis space was usually intractable. It created a projection matrix that either could not be stored in memory or iteration over the very large projection matrix was so slow that the algorithm would not converge in a reasonable amount of time. Therefore we started with linear terms and added interaction terms of increasing order (i.e. second order, third order, …) until the approximation stopped improving. We did not find an improvement by going beyond the quadratic terms.

Knot locations were explored systematically to find locations that led to good approximations. Approximations were checked in several ways. First, we plotted *v* ^*n+1*^ vs. ${\hat{v}}^{n}$ to see that they were consistent after convergence, as well as checking the variance of the residual. Second, we added knots to see if the fit was improved. Third, we increased the order of the polynomial to see if the fit was improved. Cubic polynomials (i.e. K = 3) were used in all cases. When the order was increased beyond cubic the value iteration often diverged. Finally, performance of the approximate inference MDP for the novelty task could be compared to a corresponding finite horizon model, at least for short time horizons to see if they made consistent predictions.

For the novelty task, the numerics were easier to implement if we approximated the number of samples for each option (N) and the probability that it was rewarded (p). We used a 3^rd^ order B-spline basis. Knot locations for N were 0 and 150, and the algorithm was optimized at (using Matlab colon operator notation) N = $e^{0:\frac{5}{4}:5}$ and p = 0: 0.25: 1. The N values were not integers, but this does not affect evaluation of the value function. Interactions up to second order were included. For the non-stationary two-armed bandit, the means were fit with a 3^rd^ order B-spline, and the standard deviations were fit with a 2nd order piece-wise polynomial. This approach gave well-behaved value functions. The node locations for the means were given by -30 50 and 130. The means were evaluated at 0, 12.5, 25, 37.5, 50, 62.5, 75, 87.5, 100. The node locations for the standard deviations were given by 0.25, 1, 3, 5, and 15. The standard deviations were evaluated at 0.5, 1, 2, 3, 4, 5, 7 and 14. Interactions between all basis functions up to second order were included in the model.

### Task specific details of the MPD models

#### The stationary two-armed bandit task

In the stationary two-armed bandit task one can select one of two possible bandits (actions) in each trial. Each bandit pays out with some fixed, stationary probability, and the reward amount is always the same. We modeled the task with a finite horizon, finite state, undiscounted POMDP. The state space is a discrete information state, therefore the underlying model is a discrete MDP. The state space is given by the number of times each option is chosen, and the number of times each option is rewarded, including prior information. This state space grows with trials, limiting the time horizon that can be modeled. We use lower case to indicate actual choices and rewards (r_1_, c_1_, r_2_, c_2_), and upper case to indicate posterior expected values, that incorporate prior information about choices and rewards (R_1_, C_1_, R_2_, C_2_). For a single option, assuming a beta(α,β) prior, the expected posterior reward probability, q, is $\langle q|R_{i},C_{i}\rangle \;=\;\frac{R_{i}}{C_{i}}\;=\;\frac{\alpha +r_{i}}{\alpha +\beta +c_{i}}$. The expected reward for each option under a beta(1,1) prior is $<r\left(s_{t},a\;=\;i\right)>\;=\;q\;=\;\frac{r_{i}+1}{c_{i}+2}$. State transitions are given by q. If option i is chosen, there is a transition to *R*_*i*_+1,*C*_*i*_+1 with probability *R*_*i*_/*C*_*i*_ and there is a transition to *R*_*i*_,*C*_*i*_+1 with probability *1-R*_*i*_/*C*_*i*_. Utilities for this model can be calculated using backwards induction.

#### The non-stationary two-armed bandit task

In this task there are again two bandit options. The reward delivered by each bandit is continuous, and the expected reward follows a decaying random walk. We modeled this task using an infinite horizon, continuous state, discounted, POMDP. In this case the information state is continuous, so the model is more complex than the previous POMDPs. The utilities were modeled using a basis approximation. The state space is an information space given by the current estimate of the mean and variance of the payout for each bandit [45]. We used the state transition model given in the original publication [9], except we only modeled two bandits to constrain the dimensionality of the model and make it tractable. The mean of the reward payout is given by a decaying random walk:

$$x_{i}\left(t\right)\;=\;{\lambda x}_{i}\left(t-1\right)+\left(1-\lambda \right)\theta +\eta_{x}$$ (7)

The payout is given by:

$$y_{i}\left(t\right)\;=\;x_{i}\left(t\right)+\eta_{y}$$ (8)

With $\eta_{x}\sim N\left(0,\;\sigma_{x}^{2}\;=\;7.84\right),\;\eta_{y}\sim N\left(0,\;\sigma_{y}^{2}\;=\;16\right),\;$ *λ* = 0.9836 and *θ* = 50. Both noise distributions were i.i.d. A Kalman filter can be used to track the mean for this model. The Kalman filter update for the mean and variance of the chosen option, following a reward, *y*_*i*_(*t*),is given by:

$${\hat{x}}_{i}\left(t+1\right)\;=\;{\hat{x}}_{i}\left(t\right)+\kappa_{i,t}(y_{i}\left(t\right)-{\hat{x}}_{i}\left(t\right))$$ (9)

$$\kappa_{i,t}\;=\;{\hat{\sigma }}_{i,t}^{2}/({\hat{\sigma }}_{i,t}^{2}+\;\sigma_{y}^{2})$$

$${\hat{\sigma }}_{i,t+1}^{2}\;=\;(1-\kappa_{i,t}){\hat{\sigma }}_{i,t}^{2}$$

And for the unchosen option

$${\hat{x}}_{i}\left(t+1\right)\;=\;{\lambda \hat{x}}_{i}\left(t\right)+\left(1-\lambda \right)\theta$$ (10)

$${\hat{\sigma }}_{i,t+1}^{2}\;=\;\lambda^{2}{\hat{\sigma }}_{i,t}^{2}+\sigma_{x}^{2}$$

The information state is all past observed outcomes [45]. Because the non-stationary bandit model is Gaussian, however, the sufficient statistics are given by the mean and variance of the Gaussian distribution for each bandit. Therefore the information space for this POMDP, equivalent to the state space for the MDP, can be compactly represented: $b_{t}\;=\;({\hat{\mu }}_{1},{\hat{\sigma }}_{1},{\hat{\mu }}_{2},{\hat{\sigma }}_{2})$ as the estimated mean and standard deviation (or variance) of each bandit. We do not have to retain all past outcomes, and the size of the information state space does not grow with time. For this model, the utility equation requires computation of an expectation over the observed variable given the information state. Specifically,

$$u_{t}\left(b_{t}\right)\;=\;\underset{a\in A_{s_{t}}}{\max}\left\{{\langle r\left(x_{t},a\right)\rangle }_{x}+\gamma \int_{y\epsilon S}^{}p(y_{t+1}|b_{t},a)u_{t+1}(b_{t+1}(y_{t+1}))dy\right\}$$ (11)

This integral was calculated numerically by discretizing y over +/- 2 standard deviations, and sampling 10 points. Increasing the range of y or density of sampling had minimal impact on the result. The reward expectation is given by:

$${\langle r\left(x_{t},a\right)\rangle }_{x}\;=\;\int_{}^{}r\left(x_{t},a\right)p\left(x_{t}|b_{t}\right)dx_{t}$$ (12)

which is the mean of the Gaussian. The distribution over observed values is given by:

$$p_{t}\left(y_{t+1}|b_{t},a\right)\;=\;\int_{X_{t+1}}^{}\int_{X_{t}}^{}p\left(y_{t+1}|x_{t+1},a\right)p(x_{t+1}\left|x_{t},a,b_{t}\;\right)p(x_{t}|b_{t})dx_{t}dx_{t+1}$$ (13)

Because of the linear Gaussian statistics, the integral in 16, which calculates the marginal distribution of *x*_*t*+1_ and *y*_*t*+1_ gives $y_{t+1}\sim N\;({\hat{x}}_{i}\left(t+1\right),\;{\hat{\sigma }}_{i,t+1}^{2}+{\sigma^{2}}_{y})$ and $x_{t+1}\sim N({\hat{x}}_{i}\left(t+1\right),\;{\hat{\sigma }}_{i,t+1}^{2})$ [46].

#### The novelty task

The novelty task is a 3-armed bandit task. As with the 2-armed bandit, the options are rewarded with different probabilities but the amount of reward is always 1. The reward probabilities for each bandit are stationary while that option is available. On each trial there is a 5% chance that one of the bandit options will be replaced with a new option. We have previously modeled this task with a truncated time horizon [5]. However, the exploration bonus increases with increasing time horizon. Therefore we wanted an approach that allowed us to examine longer time horizons. We used approximate methods to fit an infinite horizon, discrete state, discounted POMDP. The underlying model is, however, a discrete MDP. The state space is the number of times each option has been chosen, and the number of times it has been rewarded, s_t_ = {R_1_, C_1_, R_2_, C_2_, R_3_, C_3_}. This state space was approximated using a continuous approximation sampled discretely. The immediate reward estimate is given by the maximum a-posteriori estimate, $r\left(s_{t},a\;=\;i\right)\;=\;\frac{r_{i}+1}{c_{i}+2}$. The set of possible next states, s_t+1_, is given by the chosen target, whether or not it is rewarded, and whether one of the options is replaced with a novel option [5]. Thus, each state leads to 21 unique subsequent states. We define *qi* = *r*_*t*_(*s*_*t*_,*α* = *i*), and p_switch_ = 0.05, as the probability of a novel substitution. The transition to a subsequent state without a novel choice substitution and no reward is given by:

$$p_{t}\left({\ldots ,C}_{i}+1,R_{i},\ldots |s_{t}\;=\;[{\ldots ,C}_{i},R_{i},\ldots ],a\;=\;choose\;i\right)\;=\;{(1-q}_{i})(1-p_{switch})$$

and for reward by

$$p_{t}\left({\ldots ,C}_{i}+1,R_{i}+1,\ldots |s_{t}\;=\;[{\ldots ,C}_{i},R_{i},\ldots ],a\;=\;choose\;i\right)\;=\;q_{i}(1-p_{switch})$$

When a novel option was introduced, it could replace the chosen stimulus, or one of the other two stimuli. In this case if the chosen target, i, was not rewarded and a different target, j, was replaced, we have

$$p_{t}\left({\ldots ,C}_{i}+1,R_{i},C_{j}\;=\;0,R_{j}\;=\;0|s_{t}\;=\;[{\ldots ,C}_{i},R_{i},\ldots ],a\;=\;choose\;i\right)\;=\;{(1-q}_{i})p_{switch}/3$$

and if the chosen target was not rewarded and was replaced

$$p_{t}\left({\ldots ,C}_{i}\;=\;0,R_{i}\;=\;0,\ldots |s_{t}\;=\;[{\ldots ,C}_{i},R_{i},\ldots ],a\;=\;choose\;i\right)\;=\;{(1-q}_{i})p_{switch}/3$$

And correspondingly, following a reward and replacement of a different target, j, we have

$$p_{t}\left({\ldots ,C}_{i}+1,R_{i}+1,C_{j}\;=\;0,R_{j}\;=\;0|s_{t}\;=\;[{\ldots ,C}_{i},R_{i},\ldots ],a\;=\;choose\;i\right)\;=\;q_{i}p_{switch}/3$$

and

$$p_{t}\left({\ldots ,C}_{i}\;=\;0,R_{i}\;=\;0,\ldots |s_{t}\;=\;[{\ldots ,C}_{i},R_{i},\ldots ],a\;=\;choose\;i\right)\;=\;q_{i}p_{switch}/3$$

Note that when a novel option is substituted for the chosen stimulus, the same subsequent state is reached with or without a reward.

#### The beads task

In the beads task, the goal is to infer which of two urns is being drawn from, given a sequence of draws from one of the urns [4]. The proportion of beads in the urns is given. Beads are drawn from one of the urns, one at a time. After each bead is drawn, one can either guess that the urn with mostly blue beads is being drawn from, or that the urn with mostly orange beads is being drawn from, or one can ask to see another bead. We modeled the task as a finite horizon, discrete state, undiscounted POMDP. Because the information state space is discrete (i.e. outcomes can only be blue or orange beads) the underlying model is given by a discrete state MDP. For the current simulations the ratio of beads in the urns was always 60/40. The maximum number of beads that could be drawn was 12. The reward for being correct was 1, and the penalty for being wrong was 0, and the cost to sample was -0.005. The state space is given by the number of draws (*n*_*d*_), and the number of blue (*n*_*b*_) beads that have been drawn, *s*_*t*_ = {*n*_*d*_,*n*_*b*_}. For guessing the blue urn, a = blue, we have:

$$r\left(s_{t},a\;=\;blue\right)\;=\;C_{e}p_{o}+C_{c}p_{b}$$ (14)

where C_e_ is the cost of an error (0 for the results here) and C_c_ is the reward for being correct, which we set to 1. The probability that we are drawing from the blue urn p_b_ is given by

$$p_{b}\;=\;{\left[1+{\left(\frac{q}{1-q}\right)}^{\left(n_{d}-2n_{b}\right)}\right]}^{-1}$$ (15)

where q is the fraction of beads in the majority urn. The probability of the orange urn, *p*_*o*_, is then 1-*p*_*b*_. The second term in equation 1, which is the value of the next state, is 0 for choosing either blue or orange urns, because choosing an urn terminates the sequence. For drawing again, a = draw, we have

$$Q_{t}\left(s_{t},a\;=\;draw\;\right)\;=\;C_{s}+\sum_{j\in S}^{}p(j|s_{t},a)u_{t+1}\left(j\right)$$ (16)

where C_s_ is the cost of drawing (-0.005 unless otherwise stated). From a given state, s_t_, if one draws again, one draws either a blue or an orange bead, so the two subsequent states are, s_t+1_ = n_d_+1, n_b_+1 if a blue bead is drawn or s_t+1_ = n_d_+1, n_b_ if not. And the transition probabilities are

$$p_{t}\left(n_{d}+1,n_{b}+1{\text{|s}}_{\text{t}}\;=\;[{\text{n}}_{\text{d}},{\text{n}}_{\text{b}}],\text{a }=\text{ draw}\right)\;={\text{ qp}}_{\text{b}}+\left(1-\text{q}\right){\text{p}}_{\text{o}},\text{ and}$$

$${\text{p}}_{\text{t}}\left({\text{n}}_{\text{d}}+1,{\text{n}}_{\text{b}}{\text{|s}}_{\text{t}}=[{\text{n}}_{\text{d}},{\text{n}}_{\text{b}}],\text{a}=\text{draw}\right)=\left(1-\text{q}\right){\text{p}}_{\text{b}}+{\text{qp}}_{\text{o}}.$$

#### Patch foraging task

The state spaces for the foraging tasks are shown in Figs. 6A and 7A. We modeled the patch leaving task [19], as an infinite horizon discrete state MDP. We examined both discounted and undiscounted cases. The state space was given by the current juice level, the current travel delay, travel time (0 if not traveling), reward delay time (0 if not in the reward delay) and inter-trial interval (ITI) time (0 if not in the ITI). To simplify the state space we used a time-scale of 100 ms, and juice units of 20 µL. The ITI (1500 ms), travel (variable from 500 ms to 10,500 ms) and reward (400 ms) delays were modeled as a series of states with deterministic transitions. These are not shown explicitly in Fig. 6A. Thus, one always transitioned from ITI_1_ to ITI_2_, … to ITI_15_ (i.e. a 1500 ms ITI), and then transitioned from ITI_15_ to the choice state. There was a corresponding sequence of deterministic transitions for the travel and reward delays, both of which terminated in the ITI. The final state of the reward delay delivered a reward amount equal to the current reward. In this way, time was modeled explicitly. Reward was deterministically, not stochastically, reduced by 20 ul per choice to stay in the current patch, to simplify the model. The choice state was the only state that had more than one action available, and at that point one could stay in the current patch, or travel to a new patch. The only stochastic transition in the model was the transition to a new travel delay for the new patch, after the current patch was left. The probability of transitioning to each of the travel delays was uniform. Thus, p(travel delay = i|s_t_,a = leave patch) = 1/11, with 11 travel delays (500 ms to 10500 ms with 1000 ms intervals).

#### Foraging by sampling task

In the foraging by sampling task, subjects are shown a set of 6 gambles for each foraging bout [18]. Each gamble has a different reward associated with it. At each point, two gambles are sampled as a pair from the 6 and offered to the subjects. If the subjects reject the current pair, that pair is returned to the set, and a new pair of gambles is sampled from the set of 6. If subjects accept the gamble, they move on to a decision stage. In the decision stage subjects are told the probability that gamble 1 of the pair will pay out its reward and the probability that gamble two will pay out its reward. The subjects then have to select either gamble 1 or gamble 2 from the pair. They are then told whether or not they “won” on that trial. They then return to a new gambling bout, with 6 new individual gambles.

For this task [18], we only modeled the foraging stage. The decision stage is deterministic. It is assumed that subjects will select the option that has the highest expected value in that stage. We modeled the task using a finite horizon (60 sample max), undiscounted, discrete state MDP. The horizon could be shortened because of the cost-to-sample, but the state space for this task is small and the algorithm runs quickly. Utilities were modeled using backwards induction. The average values define the utilities in the last state (i.e. trial 60) where it is assumed the subjects would have to take the gamble on offer, and then these utilities were propagated backwards to the current trial. The state space was given by the number of combinations of gambles because at each point in the foraging stage, one pair was on offer. The 6 individual gambles leads to 15 pairs of gambles, assuming sampling without replacement from the gambles on offer, and combining symmetric pairs (i.e. gamble 2,3 is the same as gamble 3,2). Individual gambles ranged in value from 20 to 130 units in steps of 10. The cost to sample, C_s_, was set at its expected value, -7 points. From each state, subjects could choose to engage the current gamble, which led to the decision stage and the end of the foraging bout, or they could sample again, in which case they transitioned to a new gamble pair, that was sampled uniformly from the pairs of possible gambles. Thus, p(s_t_ = i|s_t_,a = sample) = 1/15. The expected value of each gamble pair was not given by the average of the two gambles, or by the max of the average value of each gamble. Rather, the expected value of a given pair was given by:

$$<r\left(s_{t}\;=\;g_{1},g_{2}\right)>\;=\;\int_{0.2}^{0.9}\int_{0.2}^{0.9}\underset{\mathrm{a\epsilon}1,2}{\max}(g_{1}p_{1},g_{2}p_{2})dp_{1}\;dp_{2}.\;$$ (17)

This is because it is assumed that the subjects will select the gamble with the highest expected value. Therefore the probabilities operate under the max operator.

#### Gittins indices and bandit tasks

It is also possible to use Gittins indices to select optimal actions in stationary bandit tasks [47]. Gittins indices (GI), or dynamic allocation indices, allow one to separate multi-armed bandit problems into a set of single armed bandit problems. The state value of each bandit is compared to a reference process. Because multi-armed bandits can be separated into single-armed bandit problems, the state space over which one calculates values is considerably smaller. However, computing the indices is more complicated for each single bandit. In addition, the indices can only be calculated over a finite horizon, so infinite horizon, undiscounted estimates cannot be obtained. Because of the simplified states, however, relatively long time horizons can be examined. Therefore, we describe here a basic calculation, based on the restart formulation given by Katehakis and Veinott [48]. We do not give any results, as they have been tabled previous [24,48]. For the stationary n-armed bandit task, the GI for state i, of a single bandit can be estimated by finding the fixed point of:

$${\text{u}}_{\text{t}}\left({\text{s}}_{\text{t}}\right)\;=\;\max_{\text{a}\in {\text{A}}_{{\text{s}}_{\text{t}}}}\left\{\text{Q}\left({\text{s}}_{\text{t}},\text{a}\right),\text{ Q}\left({\text{s}}_{\text{t}}\;=\text{ i},\text{a}\right)\right\}$$ (18)

The fixed point can be found with value iteration, as was done above. The strategy is to plug in a state, i, solve equation 18 using value iteration, and then use u_t_(s_t_ = i) as the GI for that state. This must be done for every state for which an index is required. Not that the action set here is only over the single bandit vs. the reference action, and not the set of bandits. Therefore, the indexes are valid for n bandits, once computed for a single bandit. After the GIs are calculated for all the states, one chooses the bandit whose GI is the largest, given its state in each trial. The GIs are not, however, value estimates.
